# Integrating microbiome and metabolome revealed microbe-metabolism interactions in the stomach of patients with different severity of peptic ulcer disease

**DOI:** 10.3389/fimmu.2023.1134369

**Published:** 2023-03-09

**Authors:** Chao Wang, Xiao Yu, Hongqiang Lin, Guoqiang Wang, Jianming Liu, Chencheng Gao, Mingran Qi, Dan Wang, Fang Wang

**Affiliations:** ^1^ Department of Pathogen Biology, College of Basic Medical Sciences, Jilin University, Changchun, China; ^2^ Department of Otolaryngology Head and Neck Surgery, China-Japan Union Hospital of Jilin University, Changchun, China; ^3^ Department of Gastroenterology, First Hospital of Jilin University, Changchun, China

**Keywords:** peptic ulcer, gastric flora, microbiome, metabolome, comprehensive analysis

## Abstract

**Background:**

Peptic ulcer disease (PUD) is a multi-cause illness with an unknown role for gastric flora and metabolism in its pathogenesis. In order to further understand the pathogenesis of gastric flora and metabolism in PUD, this study used histological techniques to analyze the microbiome and metabolome of gastric biopsy tissue. In this paper, our work described the complex interactions of phenotype-microbial-metabolite-metabolic pathways in PUD patients at different pathological stages.

**Methods:**

Gastric biopsy tissue samples from 32 patients with chronic non-atrophic gastritis, 24 patients with mucosal erosions, and 8 patients with ulcers were collected for the microbiome. UPLC-MS metabolomics was also used to detect gastric tissue samples. These datasets were analyzed individually and integrated using various bioinformatics methods.

**Results:**

Our work found reduced diversity of gastric flora in patients with PUD. PUD patients at different pathological stages presented their own unique flora, and there were significant differences in flora phenotypes. *Coprococcus_2*, *Phenylobacterium*, *Candidatus_Hepatoplasma*, and other bacteria were found in the flora of people with chronic non-atrophic gastritis (HC). The representative flora of mucosal erosion (ME) had *uncultured_bacterium_c_Subgroup_6*, *Sphingomonadaceae, Xanthobacteraceae*, and *uncultured_bacterium_f_Xanthobacteraceae.* In comparison, the characteristic flora of the PUD group was the most numerous and complex, including *Ruminococcus_2*, *Agathobacter*, *Alistipes*, *Helicobacter*, *Bacteroides* and *Faecalibacterium*. Metabolomics identified and annotated 66 differential metabolites and 12 significantly different metabolic pathways. The comprehensive analysis correlated microorganisms with metabolites at different pathological stages and initially explored the complex interactions of phenotype-microbial-metabolite-metabolic pathways in PUD patients at different pathological stages.

**Conclusion:**

Our research results provided substantial evidence to support some data on the analysis of the microbial community and its metabolism in the stomach, and they demonstrated many specific interactions between the gastric microbiome and the metabolome. Our study can help reveal the pathogenesis of PUD and indicate plausible disease-specific mechanisms for future studies from a new perspective.

## 1 Introduction

Peptic ulcer disease (PUD) is a multi-cause illness. PUD is closely related to *Helicobacter pylori* infection, Non-Steroidal Anti-Inflammatory Drugs, bile reflux, genetics, obesity, alcoholism, smoking and stress factors, and its pathogenesis is extremely complex (1, 2). Clinically, an ulcer is diagnosed when the mucosal rupture is equal to or greater than 5 mm in diameter and covered with fibrin; a mucosal rupture of less than 5 mm is called erosion (3). The incidence of PUD is 0.1-0.3% in the general population, occurs mainly between the ages of 25 and 64, and increases with age (1, 4, 5). The diagnosis and treatment of PUD currently remain a major healthcare problem that may consume significant social and public resources (4).

There appears to be increasing evidence that the microbiome has a vital impact in maintaining health (6–8). Notably, the microbiome has been identified as a key circumstantial factor in the pathogenesis and progression of PUD (9). However, most of the current research on gastrointestinal diseases has focused on the gut microbes and ignored the presence of specific gastric flora, and the potential mechanisms for the involvement of gastric flora are far from being explored in the development of PUD. The inflammatory process and severity of PUD may be related to structural changes in the gastric flora (10). Ecological dysbiosis of the gastric microbiota can induce a significant increase in the number and variety of opportunistic pathogenic bacteria, which can be toxic to cells and induce inflammation in the stomach (11). Bik et al. examined 128 phylotypes from human gastric biopsy samples, which were classified into five different bacterial phyla: *Proteobacteria*, *Actinobacteria*, *Firmicutes*, *Fusobacteria*, and *Bacteroidetes (* 12). The diversity of microorganisms in the gastric environment was significantly different from that in the oral cavity, esophagus, and intestine (9, 11–13). Although the mechanistic events involved in the development of PUD by *Helicobacter pylori* have been identified (6), the profile of the microbial community in the stomach at different pathological stages of PUD has not been systematically characterized. Determining the role of previously unidentified intragastric flora in the mechanisms driving the development of PUD will be clinically instructive.

Apart from the change in flora, it is increasingly recognized that other factors, such as altered metabolism in the local microenvironment, may also be involved in the pathogenesis of PUD. Metabolomics can explore the metabolic regulation between small molecule metabolites and the organism, which in turn can elucidate the pathophysiological state of the organism. In addition, it can also help researchers quickly capture information about metabolic disorders caused by overall or local dysfunction of the organism, and more easily explain complex heterogeneous diseases such as asthma (14, 15). Some of the results of Integrative Human Microbiome Project (16) confirmed that patients with gastrointestinal disorders had lower levels of secondary bile acids, vitamin B3, vitamin B5 and short chain fatty acids, but the levels of acylcarnitine and polyunsaturated fatty acids were higher. And patients with microbial ecosystem dysbiosis further promoted the development of gastrointestinal inflammation (17). Gut microbes and their metabolites can play an important role in stimulating the development of the immune system. Some Clostridial flora promote the development and maturation of organic immune organs and the immune system by producing SCFAs that stimulate the growth of regulatory T cells (18). Similarly, SCFAs boosted acetyl coenzyme A production in B lymphocytes and regulated metabolic processes to generate energy for antibody production (19).

Small molecule metabolites as intermediates or end products of microbial metabolism are intermediate mediators of microbiota-host interactions (20, 21). Microbial metabolites can influence host energy metabolism, immune homeostasis, and gastrointestinal mucosal integrity (22). Metabolomics-microbiome integration studies using a mixture of correlation and network approaches deliver a global understanding of the interactions that exist between the gut mucosa and the gut microbiome (23–25). However, the interaction between the microbiota and metabolites in the stomach has not been adequately characterized in the evolution of PUD. The study of such interactions is limited by invasive and expensive sampling techniques, and such studies usually only focus on the classical approach of a single histology (26). Few studies have been seen to focus on the interactions between gastric flora and metabolism at different stages of PUD pathogenesis, so the role of intragastric flora and metabolism in its pathogenesis is not well understood.

According to the research blank, our research systematically provided a comprehensive microbiome and metabolome profile of human gastric tissue biopsies to further characterized bacterial diversities and the abundance of metabolites, which deciphered the linkage of PUD with gastric microbiome and metabolites. In summary, this work will deepen our knowledge and comprehension of stomach flora and metabolism in the pathogenesis of PUD, which will help to further investigate the effects of flora and small molecule metabolites on PUD and thus identify new therapeutic targets or drugs for PUD.

## 2 Materials and methods

### 2.1 Patient recruitment

32 patients diagnosed with PUD or PUD development and 32 patients with chronic non-atrophic gastritis were recruited based on clinical gastrointestinal endoscopic diagnostic criteria from the First Hospital of Jilin University. They were divided into three groups: peptic ulcer disease (PUD), mucosal erosion disease (ME), and chronic non-atrophic gastritis (HC). Gastric tissue (gastric sinus mucosa) biopsy samples and medical history information were collected from all volunteers. All volunteers had no history of smoking, no history of antibiotic use within the last month, no cardiovascular, metabolic, hematological, or other diseases that affected the gastric flora, no liver or kidney dysfunction, and no recent hormone treatment. Subject volunteers were informed of the full content of this study prior to collection. The study was approved by the Ethics Committee of the First Hospital of Jilin University (AF-IRB-032-06) and registered with the Chinese Clinical Trials Registry (No. ChiCTR1800015420).

### 2.2 DNA extraction and 16S rDNA sequencing process

Gastric tissue biopsies were collected from volunteers who met the criteria, and the tissue samples were stored at -80°C. The microbial genome was extracted from the surface of gastric tissues using the MN Nucleo Spin 96 Soi DNA extraction kit. Specific primers (5’-ACTCCTACGGGAGGCAGCA-3’, 5’-GGACTACHVGGGTWTCTAAT-3’) were designed according to the conserved region (V3+V4), and the extracted genomes were amplified and purified. The quality-checked libraries were subjected to the sequencing process using the Illumina Novaseq 6000 sequencing platform.

### 2.3 Metabolomics sample preparation and UPLC-MS/MS sequencing process

Firstly, 30mg of tissue samples were weighed and mixed with 1000μL of extract solution (methanol: acetonitrile: water, 2:2:1) for 30s, followed by grinding with a 45 Hz grinder and ultrasonic crushing. The above samples were left to stand at -20°C for 2 hours, then centrifuged at 12000rpm for 15 minutes, and 500μL of supernatant was taken and dried with a vacuum concentrator. The dried extracts were dissolved with 160μL of extract solution (acetonitrile: water, 1:1) and vortexed for 30s; the samples were sonicated in a water bath and then placed in a centrifuge with the parameter setting: 12000rpm, 15min; 120μL of supernatant was placed in a 2mL injection bottle for detection. The LC-MS system for metabolomics consisted of a Waters Acquity I-Class PLUS UPLC tandem with a Waters Xevo G2-XS QTOF high-resolution mass spectrometer using a Waters Acquity UPLC HSS T3 column (1.8μm, 2.1x100mm); mobile phase A was 0.1% formic acid aqueous solution; mobile phase B was 0.1% formic acid acetonitrile. The chromatographic gradient program was: 0-0.25min, 2% B; 0.25-10min, 2%-98% B; 10-13 min, 98% B; 13-13.1min, 98%-2% B; 13.1-15min, 2% B with the flow rate at 0.4mL/min. The mass spectrometry conditions were: low collision energy of 2V, high collision energy interval of 10-40V, and scan frequency of 0.2 sec/sheet. The ESI ion source parameters were as follows: capillary voltage: 2000V (positive ion mode) or -1500V (negative ion mode); gas flow rate: 800L/h; backblast gas flow rate: 50L/h; cone hole voltage: 30V; gas temperature: 500°C; ion source temperature: 150°C.

### 2.4 Microbiome analysis

In this study, the Alpha diversity index and Beta diversity of samples were calculated and analyzed based on the QIIME2 (27) platform, and the differences between groups were assessed using a t-test. PICRUSt2 (28) and the Kyoto Encyclopedia of Genes and Genomes (29) (KEGG) were used to predict the composition and differences in metabolic pathways of the flora. Bio-coverage levels of complex microbiomes and biologically explainable phenotypes were predicted based on BugBase (30), and t-tested between different groups. The Line Discriminant Analysis Effect Size (31) (LEfSe) analysis was performed to identify key species. Spearman correlation analysis was performed in R (4.0.2), and correlation networks were constructed using the ggraph package depending on the abundance of species in each group of samples and the variation. The raw sequencing data for the microbiome had been uploaded to the BioProject section of the National Center for Biotechnology Information, and the number was PRJNA875625.

### 2.5 Metabolomics analysis

Raw metabolomics data collected using MassLynx (V4.2) were subjected to data pre-processing operations such as peak extraction and peak alignment by Progenesis QI software. Metabolite identification was performed using Progenesis QI software and METLIN database. Metabolite annotation was applied to the data based on KEGG database (29) and Human Metabolome Database (32) (HMDB). Principal Component Analysis (PCA) and Orthogonal Partial Least Squares Discriminant Analysis (OPLS-DA) were performed based on BMKCloud (www.biocloud.net). The permutation test (n=200) was used to check the stability of the OPLS-DA model. Variable Importance in Projection (VIP) reflects the contribution of the metabolite to the model variance. Enrichment analysis of identified differential metabolites (VIP>1 and P<0.05) was performed using MetaboAnalyst5.0 (33).

### 2.6 Microbiome combined with metabolomic analysis

This study used the available microbiome and metabolome data to perform Spearman correlation analysis on the bacteria screened by LEfSe and the metabolites with highly significant differences (P<0.01 and VIP>1.5), with an associated heatmap drawn based on R. Our work combined clinical information to organically unify phenotype-microbial-metabolite-pathway-host metabolic alterations and mapped Sankey diagrams to characterize these complex interactions.

## 3 Results

### 3.1 Clinical features of the patient

The subjects were divided into three groups based on endoscopic findings in this study: chronic non-atrophic gastritis (HC, n = 32), peptic mucosal erosion (ME, n = 24), and peptic ulcer disease (PUD, n = 8). Case reports containing clinical information about the subjects, including age, gender, and endoscopic diagnostic findings were collected. The summary results were listed in **Table 1**, and detailed patient information was presented in **Supplementary Material Table S1**.

**Table 1:** Statistics of clinical characteristics of subjects.

Clinical Info		HC	ME	PUD
Gender	Male	8 (25%)	9 (37.5%)	3 (37.5%)
	Female	24 (75%)	15 (62.5%)	5 (62.5%)
	P	0.2374^a^	0.6564^b^	0.3812^c^
Age	/	48.13 ± 11.62	49.13 ± 13.96	48.25 ± 7.10
	P	0.78^a^	0.97^b^	0.41^c^
Endoscopy	Non-atrophic gastritis	32	24	8
	Mucosal erosion	/	24	3
	Ulcer	/	/	8

P, the value of the t-test;/, null or not present; ^a^, HC vs. ME; ^b^, HC vs. PUD; ^c^, ME vs. PUD.

### 3.2 Changes in the gastric microbial community of patients with PUD

#### 3.2.1 Diversity of microorganisms in the stomach

**Figure 1:**
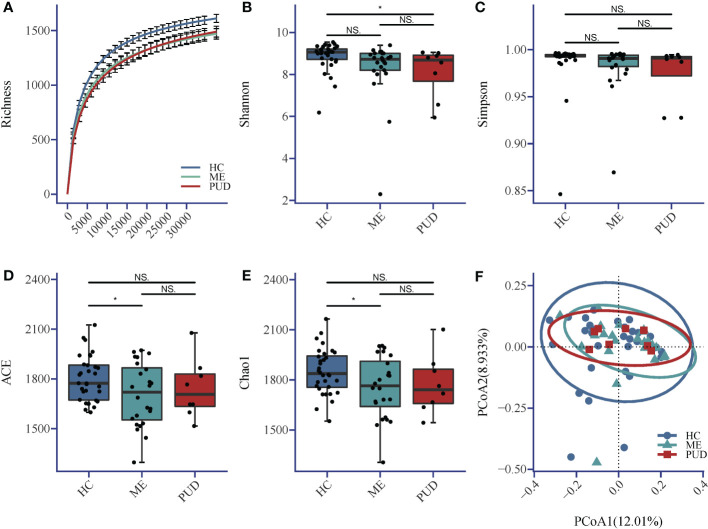
**(A)** Richness; **(B)** Shannon index; **(C)** Simpson index; **(D)** ACE index; **(E)** Chao1 index; **(F)** Beta diversity. Alpha diversity index and Beta diversity. *, a significant difference between the two groups (P<0.05); NS, no significant difference between the two groups (same below). The horizontal line in the box plot is the mean and the error bars are the standard deviation.

The sequencing depth in this research fully met the requirements for subsequent analysis (**Figure 1A**). The Shannon index (**Figure 1B**) indicated that the richness and diversity of microbial species were markedly higher in the HC group compared to the PUD group (P<0.05). The Simpson index (**Figure 1C**) showed no significant difference among the three groups. ACE index (**Figure 1D**) and Chao1 (**Figure 1E**) index showed that the richness and diversity of microbial species were significantly higher in the HC group versus the ME group (P<0.05). Beta diversity showed that the HC, ME and PUD groups had their own unique microbial community structure and distribution, and the abundance and diversity of gastric microbial communities differed in patients with different pathological stages of ulcer(**Figure 1F**). In conclusion, we were able to find that the microbial richness and diversity were significantly lower in patients with PUD (PUD group) or patients with progressive PUD (ME group), compared to patients with chronic-non-atrophic gastritis (HC group).

#### 3.2.2 Bacterial population structure and phenotype prediction

**Figure 2:**
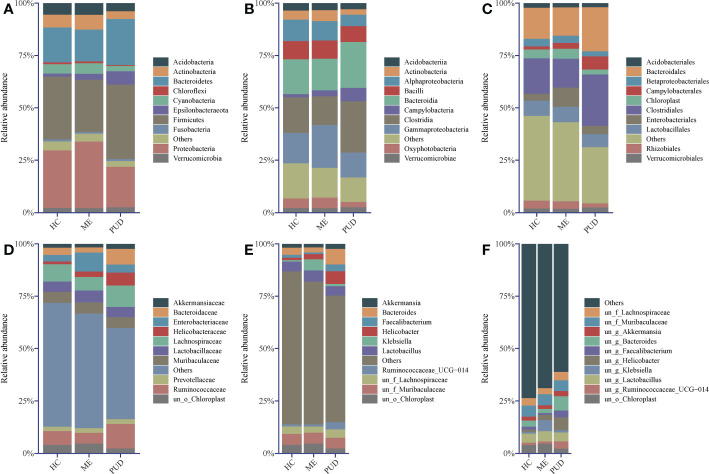
Abundance structure of top 10 flora. **(A)**, phylum level; **(B)**, class level; **(C)**, order level; **(D)**, family level; **(E)**, genus level; **(F)**, species level; un, unidentified; o, order; f, family; g, genus; others, sum of abundance of other flora.

*Firmicutes, Proteobacteria, Bacteroidetes, Actinobacteria*, and *Acidobacteria* were the primary phylum in the stomach. *Firmicutes* was the most abundant phylum in the HC and PUD, while *Proteobacteria* was the most abundant in the ME (**Figure 2A**).

In our work, the study found that the dominant class (**Figure 2B**) in the stomach included *Clostridia*, *Bacteroidia*, *Gammaproteobacteria*, and *Alphaproteobacteria*, and the amount of *Campylobacteria* in PUD was remarkably higher than that of HC and ME. From the level of the microbial community at the order (**Figure 2C**) and family (**Figure 2D**) level, found that *Campylobacterales* and even *Helicobacteraceae* caused the change in the abundance of *Campylobacteria*. The presence of *Enterobacteriaceae* bacteria in the stomach of ME patients stood out, as did their abundance and performance. Of great interest to us was the presence of *Bacteroidaceae* in higher abundance in the PUD. Our study then analyzed the genus (**Figure 2E**) and species (**Figure 2F**) and found that *Helicobacter* may be the characteristic bacterium of PUD, which is consistent with our knowledge. Moreover, we also identified some new potential pathogenic bacteria, such as *Bacteroides* and *Faecalibacterium*. In addition to this, the abundance of *Klebsiella* proved that it could be another characteristic flora of the ME. However, our present study did not allow for a more detailed classification of these genera due to the technical limitations of 16S rDNA sequencing (34). In conclusion, our work can get some important information through flora structure analysis: HC, ME, and PUD have their own particular flora constitution structure as different stages of PUD pathogenesis, which may be an important characterization in disease development.

**Figure 3:**
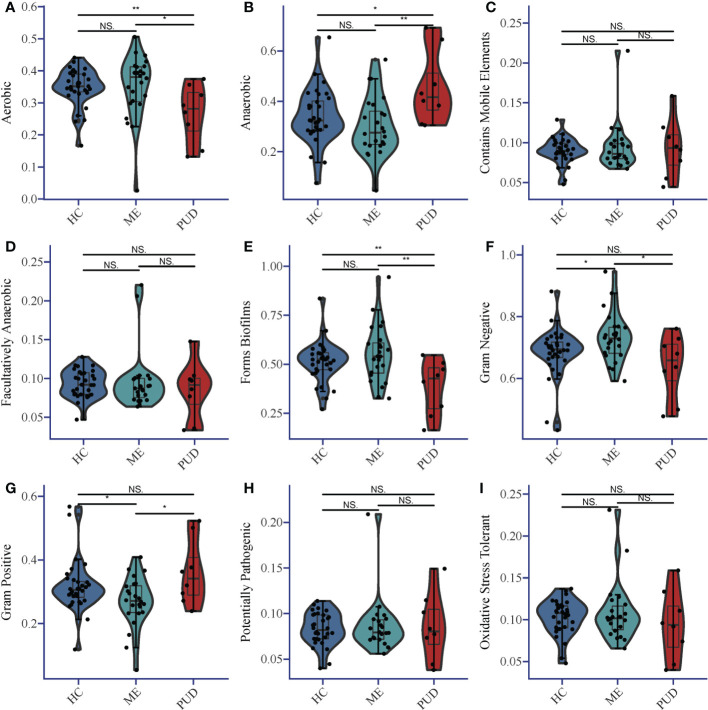
Phenotypic differences in colonies based on Bugbase prediction. **(A)**, Aerobic; **(B)**, Anaerobic; **(C)**, Contains Mobile Elements; **(D)**, Facultatively Anaerobic; **(E)**, Forms Biofilms; **(F)**, Gram Negative; **(G)**, Gram Positive; **(H)**, Potentially Pathogenic; **(I)**, Oxidative Stress Tolerant. The horizontal line in the violin plot is the mean and the error bars are the standard deviation. *, a significant difference between the two groups (P<0.05); **, a highly significant difference between the two groups (P<0.01); NS, no significant difference between the two groups (same below).

BugBase predicted the structural differences for each colony group, and **Supplementary Material Table S2** provided more information. Statistical analysis of the results indicated that the abundance of Aerobic species (**Figure 3A**) in the gastric biopsy tissues of patients with the PUD group was lower than that of the ME group (P<0.05) and extremely lower than that of the HC group (P<0.01). In opposition, Anaerobic species (**Figure 3B**) were present in higher abundance in patients with PUD as well as were significantly different from the HC group (P<0.05) and ME group (P<0.01). Forms Biofilms bacterial abundance (**Figure 3E**) was very significantly lower in samples from patients in the PUD group than in the HC (P<0.01) and ME groups (P<0.01). The ME group was diametrically opposed in Gram Negative and Gram Positive bacterial abundance (**Figures 3F, G**). Gram Negative was the highest in the ME group compared to the HC group (P<0.05) and the PUD group (P<0.05). However, the abundance of Gram Positive bacteria in the ME group was significantly lower than that in the HC and PUD groups (both P values less than 0.05). In our study, it was found that the richness of bacteria of Contains Mobile Elements (**Figure 3C**), Facultatively Anaerobic (**Figure 3D**), Potentially Pathogenic (**Figure 3H**) and Oxidative Stress Tolerant (**Figure 3I**) types did not differ remarkably in the three groups.

#### 3.2.3 Differential flora and networks of microbial relevance in the stomach

**Figure 4:**
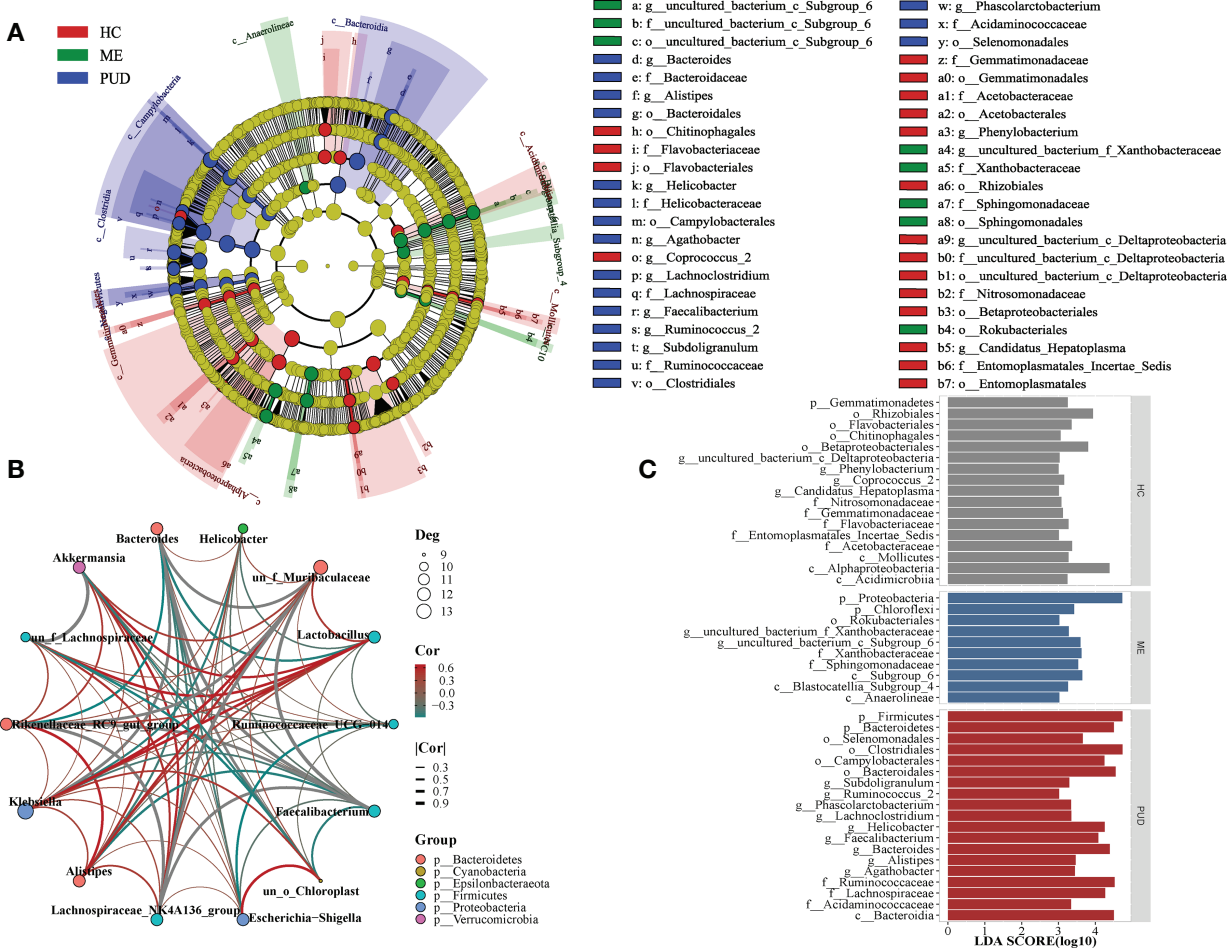
LEfSe and population correlation network. **(A)**, evolutionary tree diagram of LEfSe; **(C)**, histogram of LEfSe parameters; **(B)**, flora correlation network of PUD group. Deg indicates the number of connected nodes; Cor indicates correlation, and Group indicates the phylum to which the genus belongs.

Biomarkers of the HC, ME and PUD groups were identified by LEfSe (**Figure 4C**), and **Figure 4A** evolutionary tree showed only some of the results. In the HC group, our work discovered *Flavobacteriaceae*, *Gemmatimonadetes*, *Acidimicrobiia*, *Rhizobiales*, and *Coprococcus_2*. Ten species groups such as *Xanthobacteraceae Proteobacteria, Chloroflexi* and *Subgroup_6* could be used as biomarkers for the ME group. And the biomarkers identified from the PUD group, 19 markers including *Bacteroidia*, *Acidaminococcaceae*, *Alistipes*, *Subdoligranulum*, *Helicobacter*, and *Agathobacter*. The complete results of the LEfSe were shown in **Supplementary Material Table S3**.

Our study found different microbial community correlation network modules in the stomachs of HC, ME, and PUD patients based on the results of correlation analysis, with each of the three having its own core flora. The module of flora interactions based on the composition of *Lachnospiraceae_NK4A136_group*, *Faecalibacterium*, *Bacteroides* and *Rikenellaceae_RC9_gut_group* might be the core of the microbial correlation network in the stomach of PUD patients (under consideration of P<0.05, **Figure 4B**). *Lachnospiraceae_NK4A136_group* and *Akkermansia* can be the core group of the ME (**Figure S1**). Similarly, our study found *Akkermansia* and *Lactobacillus* to be the core group of the HC (**Figure S2**). In general, the work found that the same species were positioned differently in different groups of flora modules and that the core flora of the flora network changed gradually during the disease progression of PUD. These modules of interactions based on the composition of key species may play an important role in maintaining the microecological structure of the stomach.

### 3.3 Stomach metabolic changes in patients with PUD

#### 3.3.1 Multivariate statistical analysis of metabolomics

**Figure 5:**
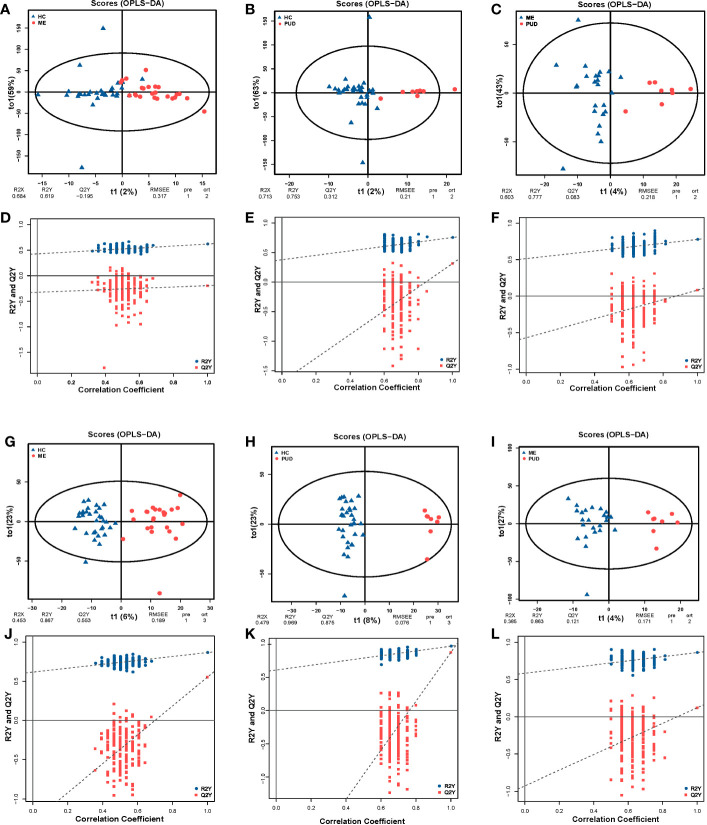
OPLS-DA and permutation test. **(A, G)**, the OPLS-DA model of HC vs. ME; **(A)**, the positive ion mode; **(G)**, the negative ion mode; **(D, J)**, the permutation test models of **(A, G)**. **(B, H)**, the OPLS-DA model of HC vs. PUD; **(B)**, the positive ion mode; **(H)**, the negative ion mode; **(E, K)**, the permutation test models of **(B, H)**. **(C, I)**, the OPLS-DA model of ME vs. PUD; **(C)** the positive ion mode; **(I)**, the negative ion mode; **(F, L)**, the permutation test models of **(C, I)**. The times of permutation tests is n=200.

PCA revealed systematic changes in metabolic disorders across various groups, with the negative spectrum of metabolites being better than the positive spectrum in terms of dispersion (**Figure S3**). The OPLS-DA models were able to separate HC, ME and PUD groups in both positive and negative modes (**Figures 5A-C, G-I**), with significant differences between the three group groups. The permutation test (n=200) revealed good stability of the OPLS-DA model construction (**Figures 5D-F, J-L**), indicating that the important variables based on the OPLS-DA model are reliable.

#### 3.3.2 Identification and screening of host differential metabolites

**Figure 6:**
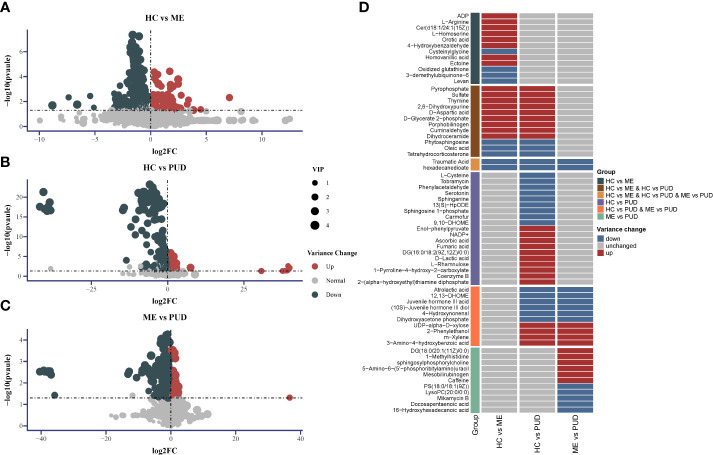
Differential metabolite Volcano and Heatmap. **(A)**, the distribution of differential metabolites for HC vs. ME; **(B)**, the distribution of differential metabolites for HC vs. PUD; **(C)**, the distribution of differential metabolites for ME vs. PUD; **(A-C)** indicates the up-regulated and down-regulated distribution of metabolites for each group of annotations; **(D)** indicates the distribution of the 66 differential metabolites in each group identified based on the KEGG database. 2-Hexaprenyl-3-methyl-5-hydroxy-6-methoxy-1,4-benzoquinone is equivalent to 3-demethylubiquinone-6.

We screened for differential metabolites using the conditions (VIP>1.0 & P<0.05), as well as metabolite characterization based on fold change. Positive ions annotated 2601 metabolites and negative ions annotated 2385 metabolites, and **Supplementary Material Table S4** provided more detailed information. There were 186 metabolites up-regulated and 252 metabolites down-regulated in the HC vs. ME (**Figure 6A**). The HC vs. PUD (**Figure 6B**) had 225 metabolites up-regulated and 280 metabolites down-regulated. The ME vs. PUD (**Figure 6C**) had 116 metabolites up-regulated and 166 metabolites down-regulated. Finally, the annotated differential metabolites were identified by comparison with the KEGG database, resulting in 66 differential metabolites (**Supplementary Material Table S5**), of which Traumatic Acid and hexadecanedioate (**Figure 6D**) showed significant differential changes in all three groups.

#### 3.3.3 Metabolism pathways of organism and flora

**Figure 7:**
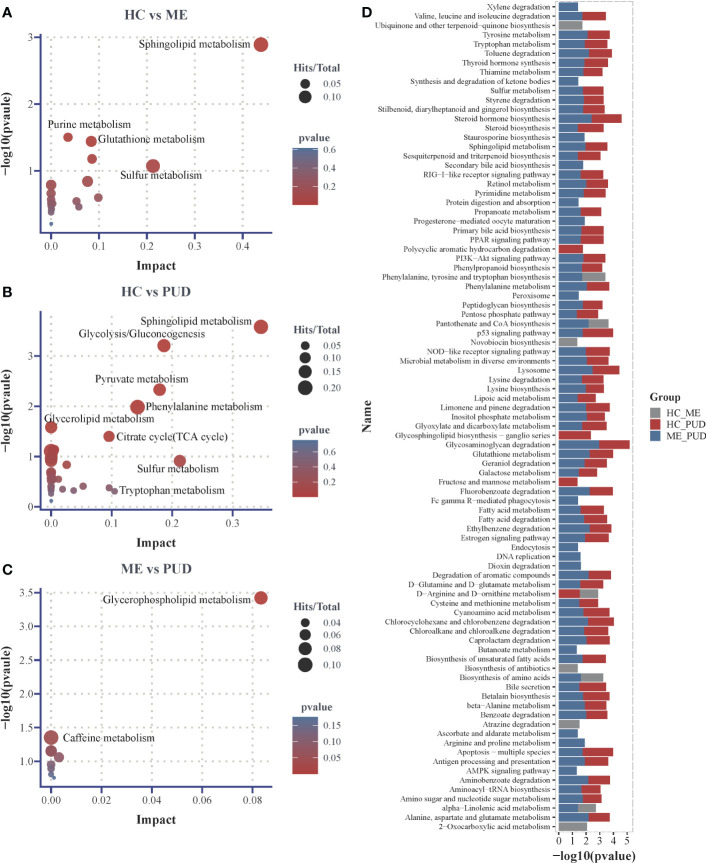
Differential metabolic pathways. **(A)**, the plot of differential metabolic pathways of HC vs. ME; **(B)**, the plot of differential metabolic pathways of HC vs. PUD; **(C)**, the plot of differential metabolic pathways of ME vs. PUD; **(D)**, the histogram of predicted metabolic pathways of the flora based on PICRUSt2 and KEGG database. Impact, the value of weight calculation based on topological analysis; larger Impact, more significant Pathway; Total, total number of metabolites in the target metabolic pathway; Hits, number of differential metabolites in the target metabolic pathway; p-value, hypergeometric distribution test.

This work believed that it may be more reasonable to characterize the perturbed metabolic pathway changes in the host based on both the hypergeometric distribution test and the topological analysis. And this study considered the pathways that were significantly perturbed as satisfying either the P<0.05 obtained by hypergeometric distribution test or Impact>0.1 obtained by topological analysis. Our research had shown that 18 metabolic pathways (**Supplementary Material Table S6**) were perturbed in the HC vs. ME group by enrichment analysis of 66 differential metabolites, with the most significant changes in Sphingolipid metabolism (P=0.0013, Impact=0.4381). Purine metabolism (P=0.0314, Impact=0.0352), Glutathione metabolism (P=0.0362, Impact=0.0838) and Sulfur metabolism (P= 0.0846, Impact=0.2123) also showed significant differential changes (**Figure 7A**). 32 metabolic pathways were perturbed in the HC vs PUD group (**Supplementary Material Table S7**). There were 4 pathways that exhibited large significant differential changes in the HC vs. PUD group, including Sphingolipid metabolism (P=0.0003, Impact=0.3469), Glycolysis/Gluconeogenesis (P=0.0006, Impact=0.1866), Pyruvate metabolism (P=0.0047, Impact=0.1794) and Phenylalanine metabolism (P=0.0104, Impact=0.1428). Glycerolipid metabolism (P=0.0261, Impact=0), Citrate cycle (TCA cycle) (P=0.0397, Impact=0.0957), Sulfur metabolism (P=0.1222, Impact=0.2128) and Tryptophan metabolism (P= 0.4911, Impact=0.1049) also showed small significant differences in variation (**Figure 7B**). ME vs. PUD group was enriched for only 9 metabolic pathways compared to the previous two groups (**Supplementary Material Table S8**), where only Glycerophospholipid metabolism (P=0.0318, Impact=0.0833) and Caffeine metabolism (P=0.0444, Impact=0) were captured with small significant differential changes (**Figure 7C**).

Our work predicted the functional composition and variability of the flora based on the PICRUSt2 and KEGG databases (**Supplementary Material Table S9**). At the primary taxonomic level, the functions of microorganisms in the stomach were mainly associated with Metabolism (approximately 78.2%), Genetic Information Processing (approximately 7.5%) and Environmental Information Processing (approximately 6.6%). At the level of secondary classification, they were mainly closely related to some metabolism, Global and overview maps (about 41.7%), Carbohydrate metabolism (about 9.3%), Amino acid metabolism (about 7.0%), Energy metabolism (about 4.3%), Metabolism of cofactors and vitamins (about 4.1%). Metabolic pathways (approximately 16.5%), Biosynthesis of secondary metabolites (approximately 7.5%), Biosynthesis of antibiotics (approximately 5.5%) and Microbial metabolism in diverse environments (approximately 4.4%) are important pathways in the tertiary classification level. With the above information, we focused on the differences of the signal transduction class pathways and metabolic pathways in the three groups. We found that 89 pathways (**Figure 7D**) were different (P<0.05), and 23 of them were highly significant (P<0.01). Glycosaminoglycan degradation (ME vs. PUD), Lysosome (ME vs. PUD), Steroid hormone biosynthesis (ME vs. PUD), Glycosphingolipid biosynthesis-ganglio series (HC vs. PUD), and Ethylbenzene degradation (ME vs. PUD) were the top five pathways with differences. Interestingly, most of these differential pathways were contributed by HC vs. PUD and ME vs. PUD.

### 3.4 The intrinsic relationship of host phenotype-flora-metabolism-pathways

**Figure 8:**
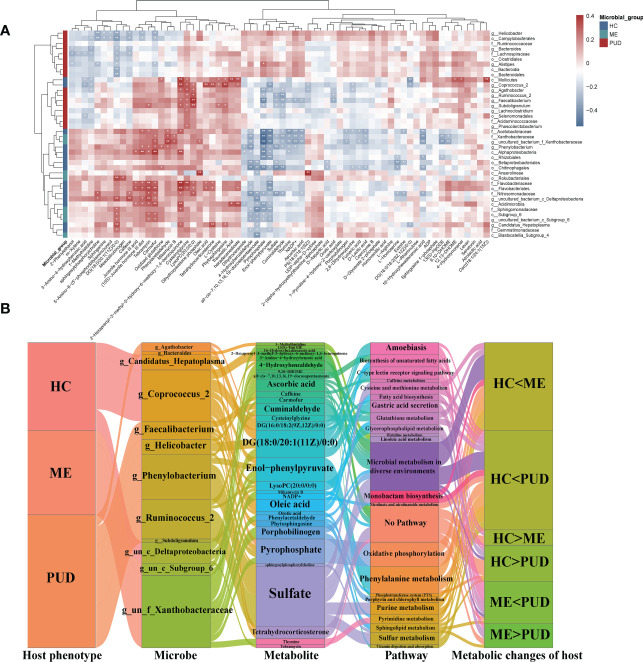
Intrinsic host phenotype-microbe-metabolite associations. **(A)**, Association of gastric flora and metabolites: heatmap of 66 identified metabolites and 46 differential flora correlation coefficients; Red-brown squares, positive correlations between these microbes and metabolites; blue-gray squares, negative correlations; the significance of statistics is indicated by “*” and “**” (*P< 0.05, **P< 0.01). **(B)**, host population-metabolite-metabolic pathway-phenotype variations, and the Sankey diagram broadly depicted host phenotypicνdifferential floraνdifferential metabolitesνmetabolic pathwaysνhost phenotypic variations; Microbe section, only the set of genera with metabolite correlations greater than 0.3; metabolite section, the set of metabolites with bacteriophage correlations greater than 0.3; Pathway section, only the host-microbe metabolic pathways and related disease pathways; g, genus; f, family; un, unidentified bacteria; “<“ and “>“, metabolite content comparisons.

This study revealed that the metabolism of the gastric flora and the metabolism of the host were closely related by predicting the metabolic functions of the flora and the enrichment of differential metabolites. In addition, a host differential metabolite-bacterial flora correlation heatmap (**Figure 8A**) was constructed to characterize the interactions between them. This work identified some possible interactions between flora and differential metabolites through correlation analysis. Overall, our work found that clustering of flora could roughly separate the PUD and non-PUD groups, while the HC and ME groups did not do so, which was similar to the results for the pathways predicted by flora. In the present study, Pyrophosphate and Enol-phenylpyruvate were significantly associated (Cor<-0.3, P<0.05) with several groups of bacteria (*Alphaproteobacteria*, *Chitinophagales*, *Rhizobiales*, *Acetobacteraceae*, *Nitrosomonadaceae*, *Sphingomonadaceae*, *Xanthobacteraceae*, *Agathobacter*, *Faecalibacterium*, *Phenylobacterium* and *un_f_Xanthobacteraceae*). Besides, *Nitrosomonadaceae*, *Agathobacte* and *Faecalibacterium* showed independent inverse correlations with Pyrophosphate. The flora was associated with some metabolite clusters (Cysteinylglycine, Traumatic Acid, Phenylacetaldehyde, 4-benzoquinone, 2-hexaprenyl-3-methyl-5-hydroxy-6-methoxy-1, PS (18:0/18:1(9Z)), Phytosphingosine, Cer (d18:1/24:1(15Z)), Dihydroxyacetone phosphate, LysoPC (20:0/0:0) and Carmofur) that had significant symbiotic relationships (Cor>0.35, P<0.005). **Supplementary Material Table S10** provided detailed information on flora and metabolism.

Our work characterized the interactions of differential genera and differential metabolism in PUD by further analysis (|Cor|>0.3 and P<0.05) of correlations that were altered metabolic pathways and host phenotypes, which also depicted some core flora and important metabolisms (**Figure 8B**). Our study focused on the role of differential genera in the flora-metabolism-host co-regulatory network. We discovered several important core genera in the stomach, including *Agathobacter*, *Phenylobacterium*, *Ruminococcus_2*, *Candidatus_Hepatoplasma*, *Coprococcus_2*, *Faecalibacterium*, and *Helicobacter*. And this study also observed some important metabolites (Sulfate, DG (18:0/20:1(11Z)/0:0), Enol-phenylpyruvate and Pyrophosphate, etc.) and some differential metabolic pathways (lipid metabolism, amino acid metabolism, gastric acid secretion and Microbial metabolism in diverse environments, and so on). Hosts of different phenotypes carried different abundance and structures of flora, which in turn could affect their own and host metabolism. These specific metabolites could serve as biomarkers to characterize altered metabolic phenotypes of the host and also had signaling effects, which could potentially affect the physiological functions of the host itself.

## 4 Discussions

There are few studies focusing on the microbial community and associated metabolism of gastric biopsy tissues of patients with peptic ulcer. In the present study, we found a profile of changes in the differential distribution of gastric flora in patients with PUD with different pathological processes. The abundance and diversity of microorganisms in the stomach decreased significantly during the development and progression of PUD, and a clinical study found that patients with chronic gastritis had a significantly higher alpha diversity index than patients with duodenal ulcers (35), which was consistent with our findings. Another clinical cohort study found that the microbiota of gastric cancer patients had lower microbial diversity than patients with chronic gastritis (36). This suggested that the abundance and diversity of our gastric flora may show a positive correlation with the health of the host, and the development of ulcers can disrupt the original microecological balance of the stomach and reduce the diversities of the gastric flora.

The dominant phyla in microbial community of the stomach were *Firmicutes*, *Proteobacteria*, *Bacteroidetes* and *Actinobacteria*, while *Acidobacteria* may be the key to differentiate the stomach flora from flora in other parts. Specialized anaerobic bacteria were the most common type of bacteria found on mucosal surfaces, and immunocompromised patients may be more susceptible to infection from anaerobic infestations (37), whereas studies had shown that probiotic mixtures containing aerobic components promote the recovery of multiple barriers in chronic colitis (38). Therefore, it was not surprising that Anaerobic flora was significantly enriched in the gastric mucosal tissue of PUD patients. Because biofilms are triggered by bacterial adhesion (39), reduced gastric flora diversity in PUD patients may be closely associated with a low abundance of biofilm-forming bacteria.

In a cohort study on ulcerative colitis, proteases from the bacterium *Bacteroides vulgatus* contributed to the activity of ulcerative colitis (40). Furthermore, *Bacteroides* and *Lachnoclostridium* may affect the levels of immunoglobulin G and component 3 in children with Henoch-Schonlein Purpura (41). The abundance of *Ruminococcus gnavus*, *Faecalibacterium*, and *Agathobacter* was significantly enriched in gut microbiome of children with allergies and asthma (42–44). All of the above were flora involved in the allergic immune response of the organism, suggesting that the emergence and progress of PUD may be strongly involved in the ecological immunity of the gastrointestinal microenvironment. Sequencing studies of slow transit constipation patients and colorectal cancer patients both identified significant enrichment of *Alistipes* and suggested that it could be used as a potential pathogenic genus and diagnostic marker for gastrointestinal diseases (45, 46). *Coprococcus_2* was significantly enriched as a probiotic in HC patients, and a clinical study of irritable bowel syndrome (47) reached similar conclusions. It had been proposed that *Subdoligranulum* may have beneficial effects on necrotizing enterocolitis in a rabbit model (48), which contradicts our findings and could be due to different diseases and species. However, *Phenylobacterium* is currently less studied in the medical field, mostly in the environment and soil, but it may also be a potential pathogenic agent of PUD.

In this study, oleic acid was strongly and positively associated with *Coprococcus_2* presentation and may act as a pancreatic lipase inhibitor in the digestive tract, reducing the digestion and absorption of fats (49). In addition, the fatty acid metabolic pathways involved in oleic acid had been linked to congenital diarrhea (50). *Bacteroides* and 1-methylhistidine had a negative correlation, which was demonstrated with a mouse model of Citrobacter rodentium-induced colitis (51), implying that altered histidine metabolism in the host or microorganism may be associated with protein histidine methylation (52). *Agathobacter*, Cysteinylglycine, and Pyrophosphate were found to have a significant correlation and, as a result, regulated the metabolic levels of Glutathione metabolism and Oxidative phosphorylation, and were involved in the progression of PUD. Cysteinylglycine was involved in energy processes as an important molecule for oxidative phosphorylation in *Actinobacteria* (53), implying that the process of damage to repair of gastric mucosal epithelial cells in PUD patients requires energy and regulates related energy metabolism *via* relevant pathways. *Phenylobacterium*, *Ruminococcus_2*, and Sulfate were found to be correlated, indicating that the flora-host regulates purine and sulfur metabolism, which is important in the pathogenesis of PUD.

*Helicobacter* was rightly linked to the development of gastric diseases (54) and was a major contributor to the development of PUD (55). But interestingly, when our study analyzed the reclassification of the sample into H group (i.e., the original HC group) and D group (i.e., the original ME and PUD groups), this later analysis found that *Helicobacter* was not the differential genus (**Figure S4**), suggesting to us that *Helicobacter* may be a characteristic flora of PUD patients while not being significant in HC and ME patients. Furthermore, *Helicobacter* had been shown to reduce the bacterial richness and diversity of the gastric flora (56), which was consistent with our findings. Relevant studies have reported the characteristics of *Helicobacter*’s physiological metabolism, including glucose metabolism, bile acid metabolism, the respiratory chain (energy metabolism), amino acid metabolism, and other pathways (57–59). Our work then focused on the metabolites associated with *Helicobacter* and the metabolic pathways that may be involved. In the present study, three metabolites, Carmofur, DG (18:0/20:1(11Z)/0:0), and sphingosylphosphorylcholine were found to be associated with *Helicobacter*, and some related important pathways were identified: Amoebiasis, C-type lectin receptor signaling pathway, and Gastric acid secretion. *Helicobacter* metabolites modified by host cholesterol had been shown to aggravate gastritis by interacting with C-type lectin receptors (60). An important metabolite, DG (18:0/20:1(11Z)/0:0), may have the same mechanism of action. A recent study had shown that *Helicobacter* sustains lives by “competing” with the body for dietary antioxidants (61) and was closely related to redox, which was consistent with the findings of this study.

Based on the above findings, we performed a more in-depth analysis and determined that the two metabolites, Traumatic Acid and hexadecanedioate, showed a trend of HC>ME>PUD in the disease process (**Supplementary Material Table S5**). Traumatic acid of plant origins had antioxidant effects in normal human fibroblasts and pro-oxidant effects in cancer cells, and was closely related to the redox response of the organism (62). Hexadecanedioate was a representative metabolite of some autoimmune diseases (63), and the dysregulation of hexadecanedioate levels also indirectly reflected a disturbance in the immune ecological balance of the stomach, perhaps an important biomarker in the PUD process. In addition, there were interesting changes in the flora: *Ruminococcus_2*, *Faecalibacterium*, *Lachnoclostridium*, *Bacteroides*, *Subdoligranulum*, and *Agathobacter* were characteristic of the PUD genera, which showed a consistent change being PUD>HC>ME. Maybe the low abundance of the gastric flora of ME was caused by the over-reaction of the immune system in mild patients(ME), which excessively cleared the gastric flora fixation. In contrast, the dysregulation of the gastric microenvironment and the local immunosuppression in severe patients (PUD) resulted in massive fixation of the flora.

Of course, this study has some limitations (64). a) The sample size of this study was small, and some clinical characteristics may lack significant correlation with the histological data, leading to the omission of some important information; b) No longitudinal study was conducted in this study, and consecutive tissue samples from recruited patients were not available; c) and diet can severely affect the microbiome and metabolome of the stomach, but we were unable to obtain information on the patient’s diet; d) In this study, due to technical limitations, the 16S rDNA sequencing could not identify the species information of the flora for more accurate identification of bacterial genera, and some functional information may not be captured. Functional studies are necessary for our future work to dissect the underlying mechanisms. Therefore, in future studies, more quasi-group macrogenomics and targeted metabolomics studies using multiple centers and large sample sizes of clinical cohort studies will be needed to validate our findings.

Even so, our results mapped the complex interplay of phenotype-microbial-metabolite-metabolic pathways in patients with different pathological stages of PUD, opening up new areas of interest regarding the association between the gastric microbiome and the metabolome of gastric biopsy tissue and PUD. In conclusion, our study will provide some data to support the analysis of microbial communities and their metabolism in the stomach as well as provide a new avenue to explore host-microbiome-metabolome associations for biomarker discovery.

## 5 Conclusions

To the knowledge of us, our study may be the first to characterize the altered microbiome-metabolome profiles of PUD patients at different pathological stages and to identify unique phenotype-microbial-metabolite-metabolic pathways interactions. Our research results provided substantial evidence to support some data on the analysis of the microbial community and its metabolism in the stomach, and they demonstrated many specific interactions between the gastric microbiome and the metabolome. Our study can help reveal the pathogenesis of PUD and indicate plausible disease-specific mechanisms for future studies from a new perspective.

## Data availability statement

The raw sequencing data for the microbiome had been uploaded to the BioProject section of the National Center for Biotechnology Information, and the number was PRJNA875625. Clinical information and raw data of the metabolome can be found in the **Supplementary Material**.

## Ethics statement

The studies involving human participants were reviewed and approved by the Ethics Committee of the First Hospital of Jilin University (AF-IRB-032-06). The patients provided their written informed consent to participate in this study.

## Author contributions

Conceptualization, CW and FW; methodology, CW, XY and HL; software, CW, XY, JL, and HL; validation, CW, GW, CG and MQ; formal analysis, CW, JL and XY; resources, FW and DW; data curation, CW, FW, and DW; writing-original draft preparation, CW and GW; writing-review and editing, FW and DW; visualization, CW; supervision, FW; project administration, DW and FW; funding acquisition, DW and FW; All authors contributed to the article and approved the submitted version.
