# Supplementary figures and images for: Integrating microbiome and metabolome revealed microbe-metabolism interactions in the stomach of patients with different severity of peptic ulcer disease
Source: Front Immunol. 2023 Mar 9;14:1134369. doi: 10.3389/fimmu.2023.1134369 (PMC10034094; doi:10.3389/fimmu.2023.1134369)

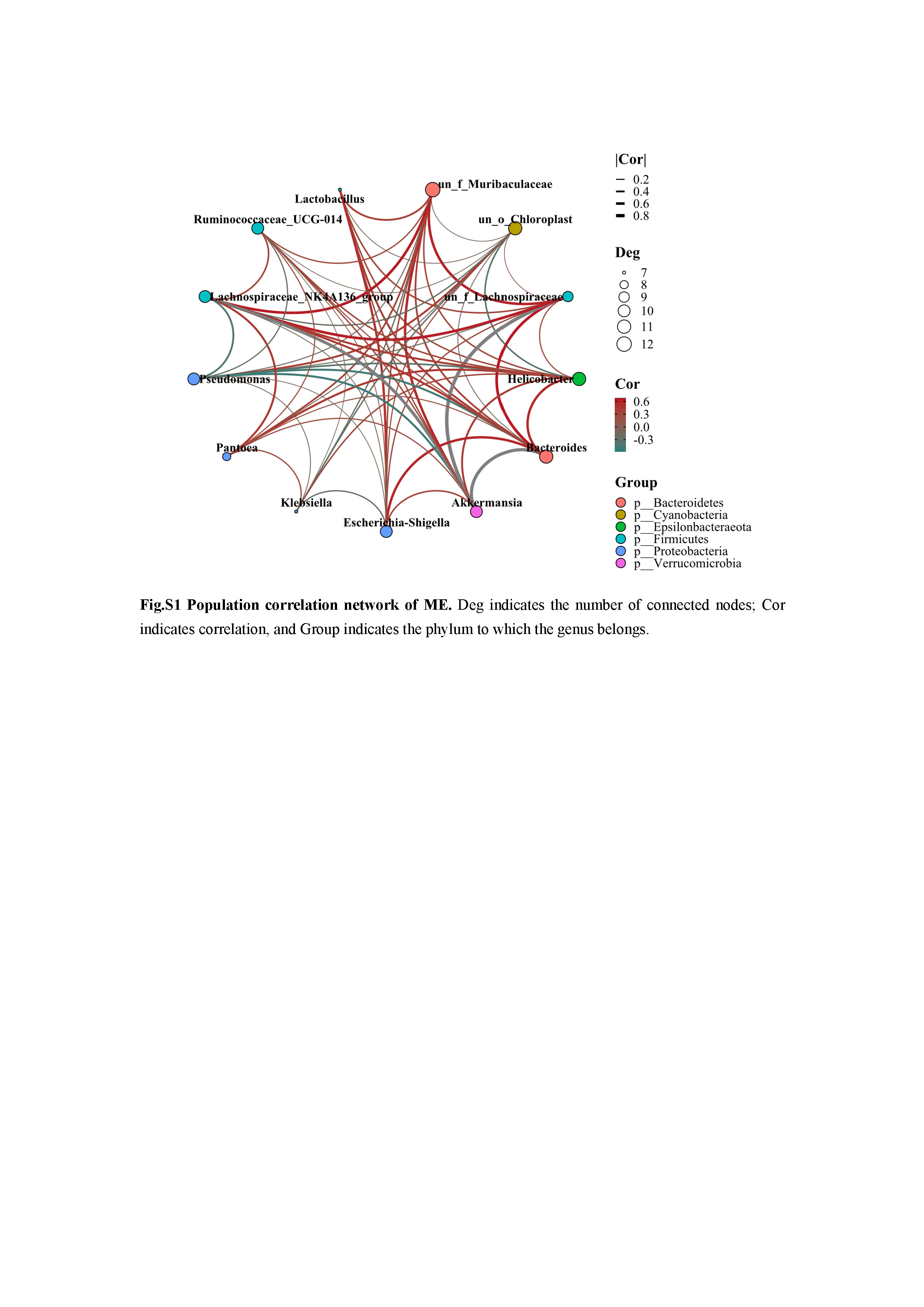

Supplement: Supplementary file 1 [file Image_1.tif]

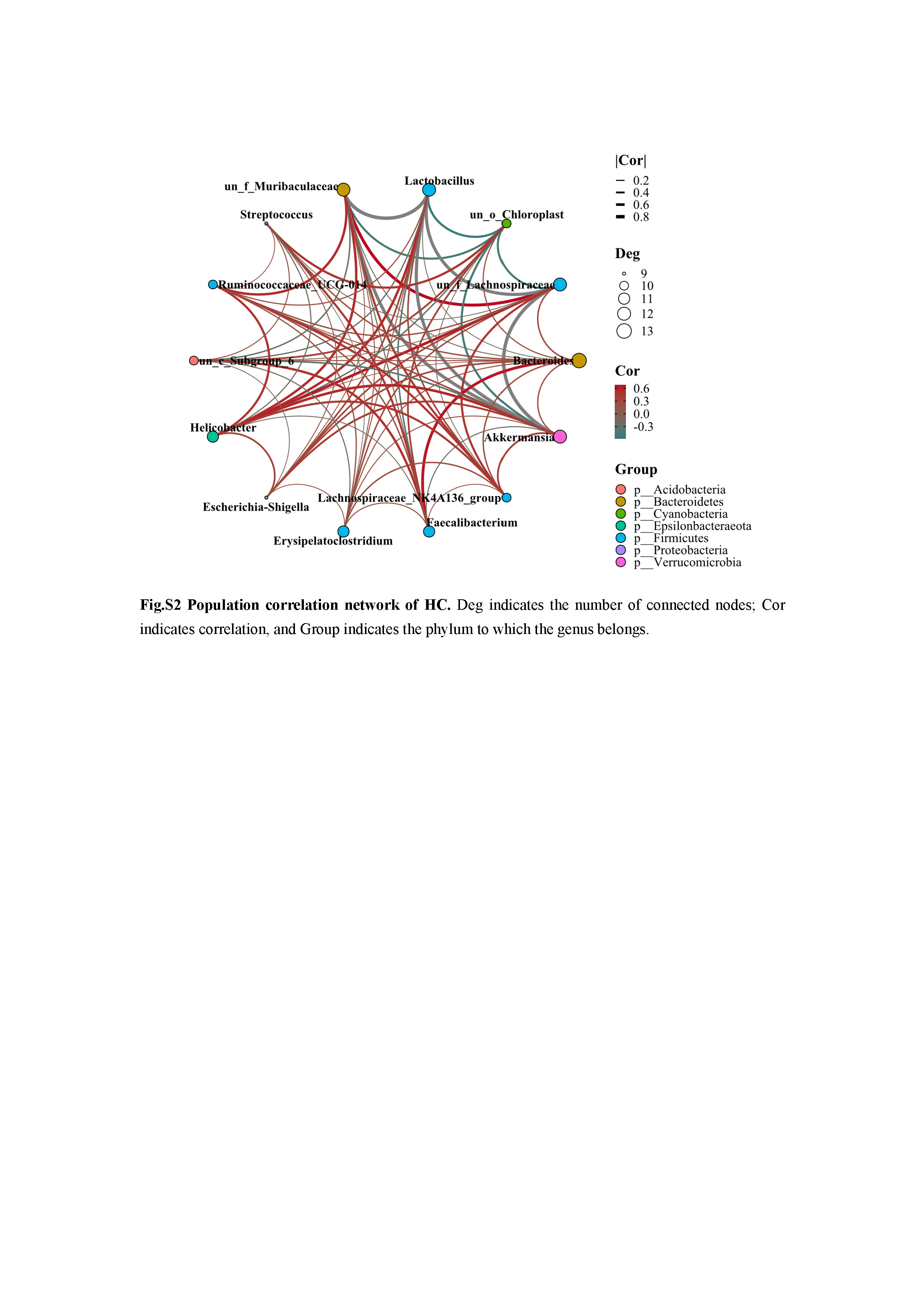

Supplement: Supplementary file 2 [file Image_2.tif]

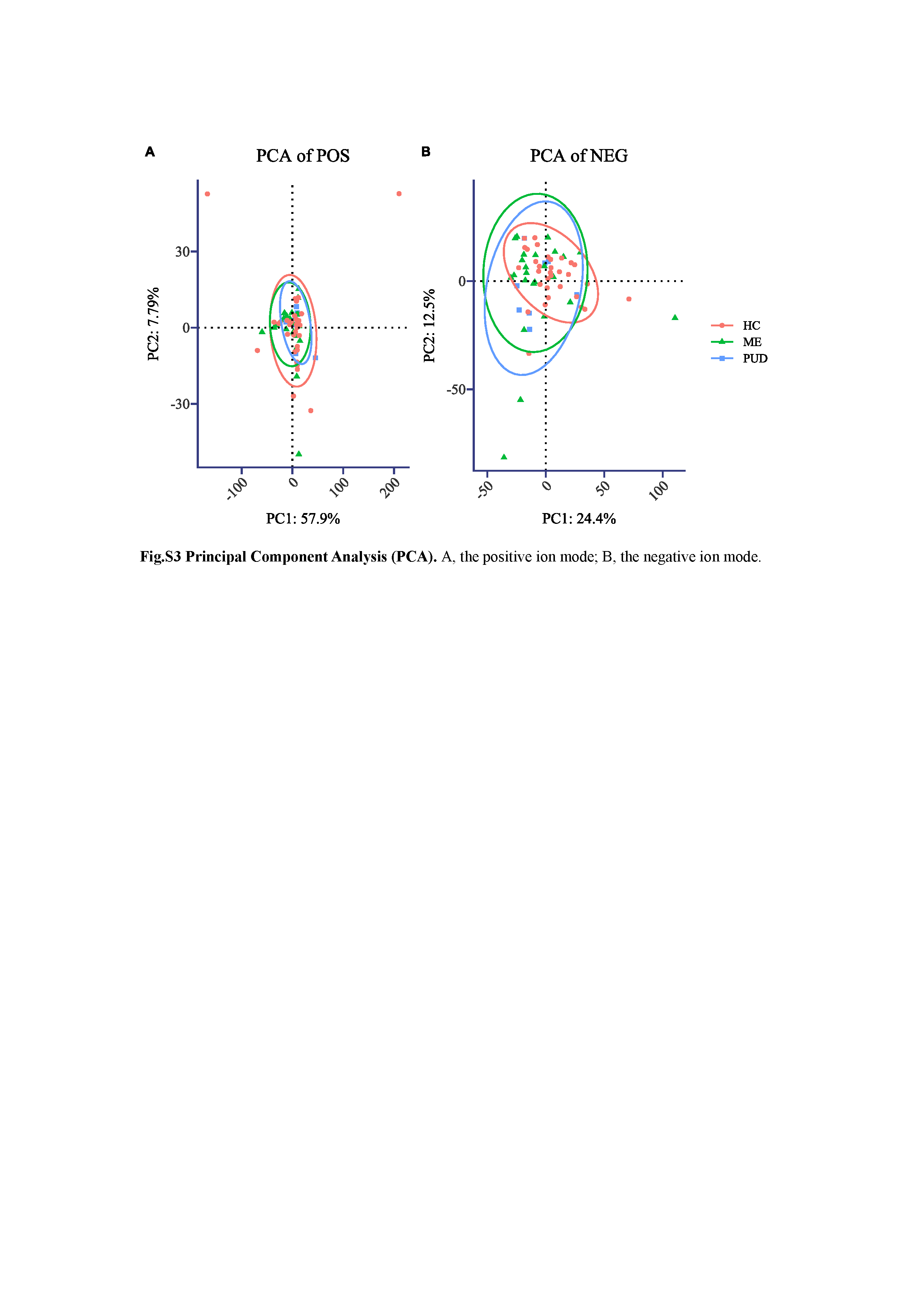

Supplement: Supplementary file 3 [file Image_3.tif]

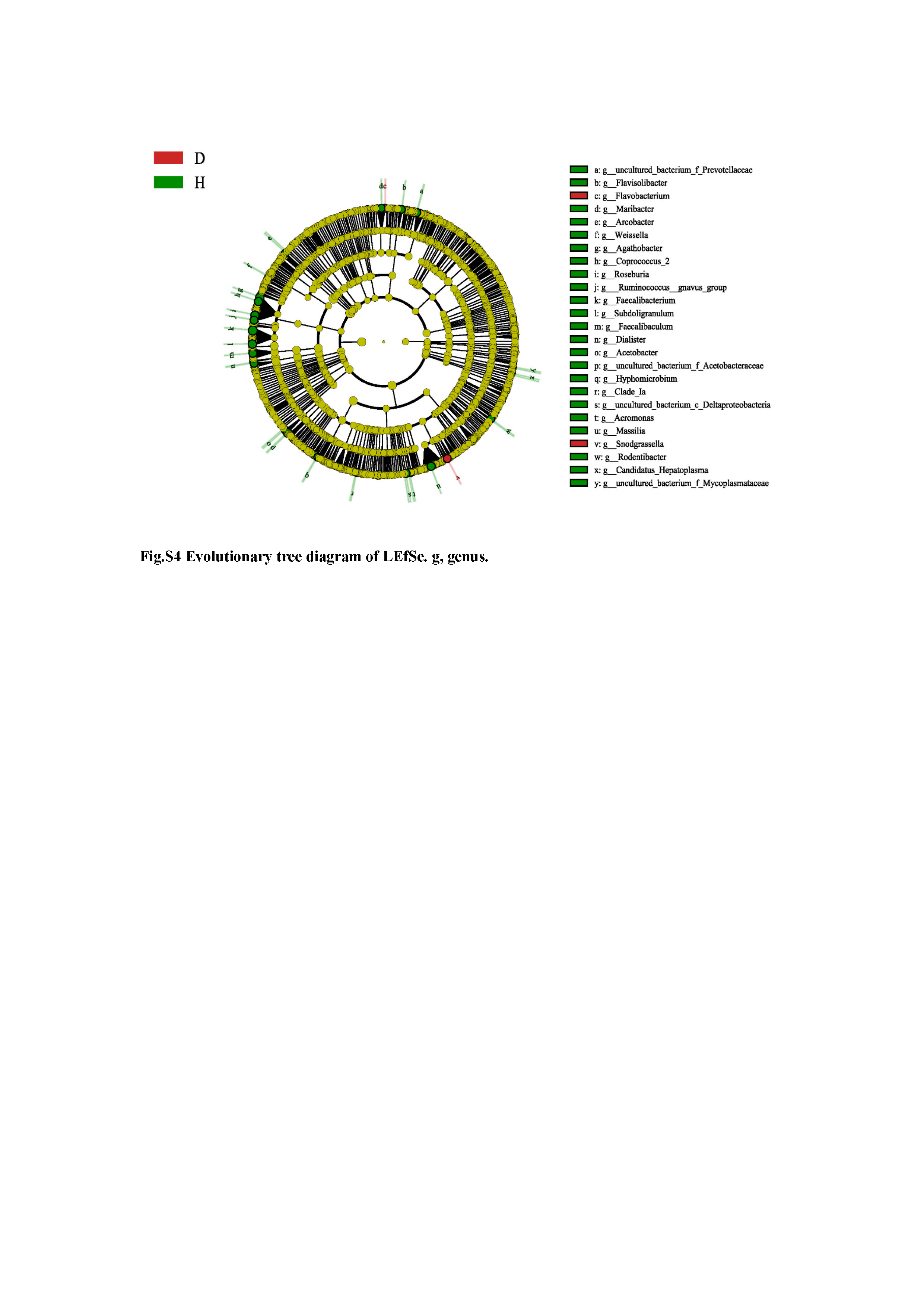

Supplement: Supplementary file 4 [file Image_4.tif]
